# Complexity of pathomechanisms leading to diastolic heart failure in diabetes mellitus - potential field for therapeutic interventions?

**DOI:** 10.1186/s12872-017-0688-x

**Published:** 2017-09-21

**Authors:** Michael Schwarzer, Michel Noutsias, Frank Spillmann, P. Christian Schulze, Torsten Doenst, Carsten Tschöpe

**Affiliations:** 1 Department of Cardiothoracic Surgery, University Hospital Jena, – Friedrich-Schiller-University Jena, Am Klinikum 1, D-07747 Jena, Germany; 2 Department of Internal Medicine I, Division of Cardiology, Pneumology, Angiology and Intensive Medical Care, University Hospital Jena, Friedrich-Schiller-University Jena, Am Klinikum 1, D-07747 Jena, Germany; 3 Department of Cardiology, Charité - Universitätsmedizin Berlin, Campus Virchow Klinikum (CVK), Berlin, Germany; 4 Deutsches Zentrum für Herz Kreislaufforschung (DZHK) – Standort Berlin, Charité - Universitätsmedizin Berlin, Campus Virchow Klinikum (CVK), Berlin, Germany; 5 Berlin Center for Regenerative Therapies (BCRT), Campus Virchow Klinikum (CVK), Berlin, Germany

## Abstract

Advanced glycation end products (AGE) have been implicated in diabetes associated complications. They have been suggested as potential mediators in the progression of diabetic heart failure and as a potential target for treatment. Brunvand et al. now provided evidence in that the suggested causal relationship between AGE and diastolic myocardial dysfunction cannot be confirmed in children with type 1 diabetes. The early signs of diastolic myocardial impairment were associated with higher BMI, but not with HbA1c levels. Furthermore, higher serum levels of MG-H1 and increased arterial stiffness were not significantly associated with diastolic dysfunction. The lack of association argues against an essential role of AGEs. This sobering finding does not support the potential to treat diastolic dysfunction by reduction approaches AGE in type 1 diabetic patients. Further pathogenic mechanisms involved in diabetic cardiomyopathy, such as alterations of calcium metabolism, or remodeling of the extracellular matrix, and intramyocardial inflammation may be further promising therapeutic targets.

Diabetes is associated with a high risk of developing heart failure. High glucose levels have been suggested as major factor in the development of comorbidities, which have a substantial adverse prognostic impact. High glucose levels carry the risk of non-enzymatically reacting with proteins to form advanced glycation end products (AGE). These AGEs have been implicated in diabetes-related complications [1]. Multiple investigations have shown an association between increased AGE and arterial stiffness and/or impaired ventricular function. However, it is not known whether AGEs are causally related to diabetic comorbidities. Already in young type 1 diabetic patients without complications, increased AGE plasma levels have been reported [2]. Furthermore, plasma AGE levels have been associated with elevated hemoglobin A1c and indices of membrane alterations [3]. AGE has also been suggested as an environmental risk factor for the development of type 1 diabetes [4].

Brunvand et al. investigated, to the best of our knowledge, for the first time the association of AGEs with early diastolic dysfunction in type 1 diabetic patients [5]. The study confirmed an association of an early loss of diastolic function and BMI, higher systolic blood pressure and higher diastolic blood pressure. However, there was no association between HbA1c or AGE levels and diastolic function in a logistic regression model. As a consequence, Brunvand et al. suggest that the diastolic dysfunction may be partly caused or initiated by factors present before the start of insulin treatment, and may thus not necessarily be reflected by HbA1c levels. Thus, the current investigation challenges one current major hypothesis of a substantial pathogenic role of AGE as a major contributor for the development of diastolic dysfunction in Type 1 diabetes patients.

With regard to the methodology of this report, we should consider that several parameters should be confirmed for the echocardiographic evaluation of diastolic heart failure [6]. Nonetheless, the authors have previously shown that the sole parameter used in their investigation, namely E’/A’-ratio < 2.0, shows a high diagnostic reliability [7]. Addressing the data conveyed by the publication, we propose that further multiple pathomechanisms than the ones studied in this report might be more relevant for the development of diastolic dysfunction in Type 1 diabetes, which ultimately might also be true for Type 2 diabetes patients. These include oxidative stress [8]. Impaired nitric oxide (NO) production affects endothelial repair mechanisms leading to endothelial dysfunction and to increased endothelial permeability [9, 10]. Hyperglycemia induces reactive oxygen species (ROS) production, contributing to endothelial dysfunction, which precedes the manifestation of atherosclerosis [11]. Increased ROS induce inflammatory transcription factor (NF-kB) activation [10], which may ultimately also contribute to intramyocardial low-level inflammation in diabetic cardiomyopathy, accompanied by a compromise of hemodynamic parameters, including impairment of diastolic function parameters [12]. Myocardial inflammation might be profoundly involved in the significant remodeling in diabetic cardiomyopathy [13]. Since these pathogenic mechanisms have been effectively blunted in experimental diabetic cardiomyopathy, including the improvement of defective sarcoplasmic reticulum Ca^2+^ transport [14], they appear a promising field to address mechanisms which effectively might prevent progression of diabetes related cardiovascular complications. The AGEs instead, appear no longer a promising target for the prevention of Type 1 and Type 2 diabetic complications.

It may be concluded that the current report by Brunvand et al. “resets” our current understanding of the relevant pathomechanisms of diastolic dysfunction in diabetes mellitus. AGEs may not be causally involved, eliminating also a causal treatment option, in this regard. Thus, the search continues, and based on the above-mentioned possibilities, at least the future outlook “is still sweet”.
